# Antimicrobial Potential, Identification and Phylogenetic Affiliation of Wild Mushrooms from Two Sub-Tropical Semi-Evergreen Indian Forest Ecosystems

**DOI:** 10.1371/journal.pone.0166368

**Published:** 2016-11-30

**Authors:** Lallawmsanga, Ajit Kumar Passari, Vineet Kumar Mishra, Vincent Vineeth Leo, Bhim Pratap Singh, Geetha Valliammai Meyyappan, Vijai Kumar Gupta, Sivakumar Uthandi, Ramesh Chandra Upadhyay

**Affiliations:** 1 Molecular Microbiology and Systematics Laboratory, Department of Biotechnology, Aizawl, Mizoram University, Mizoram, India; 2 Molecular Glyco-biotechnology Group, Department of Biochemistry, National University of Ireland Galway, Galway, Ireland; 3 Department of Agricultural Microbiology, Tamil Nadu Agricultural University, Coimbatore, Tamil Nadu, India; 4 Directorate of Mushroom Research, ICAR, Solan, Himachal Pradesh, India; Banaras Hindu University, INDIA

## Abstract

The diversity of wild mushrooms was investigated from two protected forest areas in India and 231 mushroom specimens were morphologically identified. Among them, 76 isolates were screened for their antimicrobial potential against seven bacterial and fungal pathogens. Out of 76 isolates, 45 isolates which displayed significant antimicrobial activities were identified using ITS rRNA gene amplification and subsequently phylogenetically characterized using random amplified polymorphic DNA (RAPD) and inter-simple sequence repeat (ISSR) markers. Sequencing of the ITS rRNA region classified the isolates into 16 genera belonging to 11 families. In total, 11 RAPD and 10 ISSR primers were selected to evaluate genetic diversity based on their banding profile produced. In total 337 RAPD and 312 ISSR bands were detected, among which percentage of polymorphism ranges from 34.2% to 78.8% and 38.6% to 92.4% by using RAPD and ISSR primers respectively. Unweighted Pair-Group Method with Arithmetic Mean (UPGMA) trees of selected two methods were structured similarly, grouping the 46 isolates into two clusters which clearly showed a significant genetic distance among the different strains of wild mushroom, with an similarity coefficient ranges from 0.58 to 1.00 and 0.59 to 1.00 with RAPD and ISSR analysis respectively. This reporthas highlighted both DTR and MNP forests provide a habitat for diverse macrofungal species, therefore having the potential to be used for the discovery of antimicrobials. The report has also demonstrated that both RAPD and ISSR could efficiently differentiate wild mushrooms and could thus be considered as efficient markers for surveying genetic diversity. Additionally, selected six wild edible mushroom strains (*Schizophyllum commune* BPSM01, *Panusgiganteus* BPSM27, *Pleurotus*sp. BPSM34, *Lentinus*sp. BPSM37, *Pleurotusdjamor* BPSM41 and *Lentinula* sp. BPSM45) were analysed for their nutritional (proteins, carbohydrates, fat and ash content), antioxidant potential. The present findings also suggested that the wild edible mushroom strains do not have only nutritional values but also can be used as an accessible source of natural antioxidants.

## Introduction

Mushrooms are fleshy fungi and have become attractive as a source for the development of several extracellular enzymes with agricultural and biotechnological significance. Worldwide, they are well recognized as supplementary food due to their high nutritional values [1, 2] and medicinal importance, which includes their antioxidant and antimicrobial activities [3, 4], immune enhancer [5–7], and to be effective for the treatment of diabetic [8, 9] and few types of cancers as well [10,11].

Even today, most of the tribal people in rural areas use wild mushrooms as their mainstay which sometimes leads to fatality due to the lack of knowledge in differentiating between edible and non-edible/poisonous mushrooms [12]. Moreover, their ecological influence also varies from one to another depends on their lifestyle [13]. In this context, there is a need to scientifically characterize and document the wild fruiting bodies, especially from the tribal regions and also to explore their potential for sustainable development.

Currently, amplification of the Internal Transcribed Spacers region of ribosomal DNA (ITS rDNA) has been proven as a powerful tool for the identification and phylogenetic analysis of mushrooms [14–18]. In this study, we selected two DNA based markers like randomly amplified polymorphic DNA (RAPD), and inter-simple sequence repeats (ISSR) to understand the genetic diversity among the wild mushrooms as these marker systems have been routinely employed to assess genetic diversity [19,20].

Northeastern India is well known for its rich biodiversity and for its un-tapped bioresources which fallsunder Indo-Burma Mega biodiversity Hot Spot [21]. Though, few studies have been carried out in respect to the nutritional values and industrial applications of wild and edible mushrooms fromNortheast India [22–24]. However, there are no published reports on the collection and application of wild mushrooms from protected forest areas of Mizoram, Northeast, India. Moreover, urbanization, population growth, deforestation and shifting cultivation have led to loss of biodiversity in the state [25]. At the same time, wild mushroom collection based on their traditional knowledge is an alternative source of their income in the region studied. The tribes of this region collect the wild naturally growing mushrooms, commonly known as “*Pa*” and sold them at local market for the betterment of their family. Hence, the present work was focused on the collection of wild fruiting bodies from two protected forest areas of Mizoram, Northeast India and was morphologically and molecularly identified by the amplification of the Internal Transcribed Spacer (ITS) region of ribosomal RNA (rRNA). Mycelial cultures were raised and screened for their antimicrobial activities. Furthermore, the identified mushrooms were genetically characterized by using RAPD and ISSR molecular markers. All the collected specimens were deposited in the Directorate of Mushroom Research, Solan, India. We hypothesized that the present study in general will help the local mushroom hunters to differentiate between edible and poisonous fruiting bodies. Overall the selected potential isolates can be exploited for the development of effective antimicrobials.

## Materials and Methods

### Study areas

**Fig 1:**
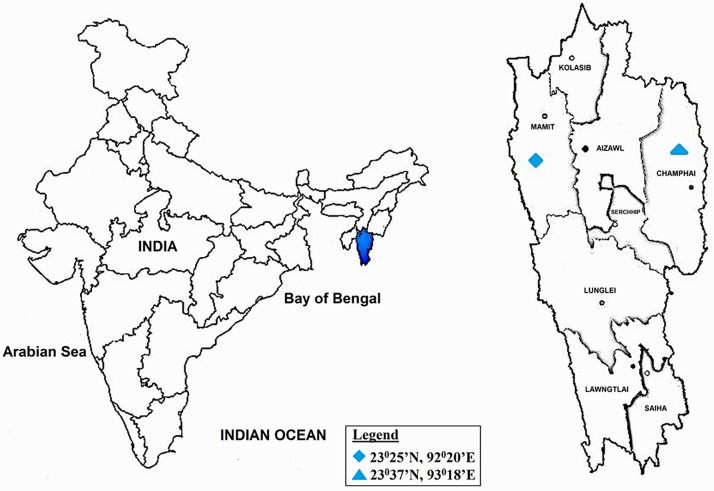
Map revealed the locations of the Sub-tropical Semi-evergreen forest ecosystems sampling sites of Dampa TR and Murlen NP, Mizoram, India.

The following protected forest areas were investigated inthe present study:

1. Dampa Tiger Reserve (DTR) (23° 23'N—23° 42’N; 92° 16'E—92° 25'E), which is one of the largest wildlife sanctuaries in Mizoram. It covers an area of ≈ 500 km^2^ (Fig 1) and the altitude of the reserve is from 800 to 1100 m above sea level with annual rainfall during the years of sampling ranges from 2000 mm to 2500. Maximum rainfall was observed mostly during June to August every year. The winter (October–January) is a cool dry season with few rainy days. Summer (February to May) is largely hot and dry, with occasional thundershowers and pre-monsoon rains in April-May.It has fine loam type of soil which covers over 48% of the total area of the state. The soil pH is ≈5.34 and is rich in Nitrogen (N), Phosphorus (P) and Potassium (K). Temperature ranges from 12°C to 25°C during winter and between 22°C to 35°C during summer. DTR is recognised as sub-tropical semi-evergreen.
2. Murlen Nation Park (MNP) (23° 32'—23° 41'N; 92°13'—92°27'E) is located close to China Hills and covers an area of ≈ 100 km^2^ (Fig 1). Altitude ranges from 1,000–1,600 m and the average annual rainfall is about 2500 mm. MNP is classified as sub-tropical semi-evergreen and sub-montane forest. Winter is usually from October to mid-February and summer is from February/March to May. The pH of the soil is ≈5.92 and the soil type is mostly clayey fine soils which are usually deep and well drained.The temperature during winter ranges from 4°C to 20°C and 22°C to 32°C during summer.

### Collection of wild mushrooms

Selected forests were visited for three consecutive years (2013–15) during rainy and winter sessions (June to December). Permission to collect wild mushrooms from the protected forests was obtained from the Chief Wildlife Warden, Environment and Forest Department, Government of Mizoram, India issued by Mr. LiankimaLailung, Conservator of Forest (WL), Mizoram, India. The wild mushrooms were identified *in situ* if possible or else the specimens were retained for later identification. The samples were collected in sterile paper bags and brought into the laboratory. Morphological parameters (pileus, lamellae, stipe, spore print and color) were assessed from fresh samples as per Largent and Stuntz [26]. Digital pictures were taken at natural habitat as well as under laboratory conditions and characterized morphologically up to genus level.

### Tissue culture of wild mushrooms

The collected fruiting bodies were used to raise mycelial culture as per the modified method of Bolla et al. [27]. Briefly, the collected fruiting bodies were cleaned thoroughly by using fabric paint brush. A small piece of tissue from the fruiting body was dipped in 0.05% sodium hypochloride (NaOCl) solution and washed three times with sterile distilled water. The samples were dried on sterilized tissue paper and transferred aseptically on to potato dextrose agar (Hi-Media, Cat No: MH096) media and incubated at 28°C for 5–7 days.

### Test organisms for antimicrobial activities

Antimicrobial activities were tested using three bacterial pathogens (*Staphylococcus aureus*: MTCC-96; *Escheridia coli*: MTCC-739; *Pseudomonas aeruginosa*: MTCC-2453), three fungal pathogens (*Fusarium oxysporum*: MTCC-2946; *Fusarium proliferatum*: MTCC-286; *Fusarium oxy* f. sp. *ciceri*: MTCC-2791) and a yeast pathogen (*Candida albicans*: MTCC-3017). All the tested pathogens were obtained from the Microbial Type Culture Collection (MTCC), Chandigarh, India and were maintained at Molecular Microbiology and Systematics Laboratory, Department of Biotechnology, Mizoram University on specific media as per instructions.

### Evaluation for antifungal potential

All the isolates were screened for their *in vitro* antagonistic activity by dual culture technique [28, 29]. Briefly, one 5 mm agar block of fully grown fungal pathogen was placed at the centre of the petridish containing PDA, and mushroom isolates were kept at the periphery of the plates. Petri dishes were incubated at 28°C for 7 days and diameter of the fungus growth was measured as compared to control. All the experiments were carried out in triplicate and the percentage of inhibition was calculated using the formula: C-T/C×100; Where, C is the fungal pathogen control and T is the distance of the tested pathogen with the sample.

### Evaluation for antibacterial potential

Antibacterial activity was determined by modified agar well diffusion method [30]. Briefly, an aliquot of the tested bacterial pathogens (50 μl) were spread on the surface of the modified LB agar plates using sterile L spreader and well of 6 mm diameter was prepared by using sterile cork borer. The wells were filled with 100 μl (1×10^−4^ CFU/ml) of cell free extractof mushroom isolate and the plates were incubated at 37°C for 24 h. All experiments were carried out in triplicates and the zone of inhibition was measured in mm.

### Molecular characterization of Mushrooms

#### DNA extraction and ITS-rRNA gene amplification

Total genomic DNA was extracted from the freshly grown mycelial mat as described by Cenis [31] with minor modification. The internal transcribed spacer (ITS) region of the rDNA was amplified using universal primers ITS1 (5’-TCCGTAGGTGAACCTGCGG-3’) and ITS4 (5’-TCCTCCGCTTATTGATATGC-3’) [32]. PCR reaction mixture was carried out in a total volume of 25 μL containing, 1X PCR assay buffer with 1.5 mM MgCl_2_, 2.5mm dNTPs, 10 pmols of each primer, 50 ng of template DNA and 1 unit of Taq DNA polymerase. The PCR was carried out in the Applied Biosystems thermal cycler with an initial denaturation at 95°C for 5 min, followed by 30 cycles of denaturation at 95°C for 1 min, annealing at 57°C for 1 min, extension at 72°C for 2 min and a final extension at 72°C for 7 min. The amplified PCR product was separated on 1.5% agarose gel and visualized under Bio-Rad Gel Documentation System XR+ (Hercules, CA, USA). The products were purified by using the Pure-link PCR Purification Kit (In-vitrogen) and sequenced commercially at Sci-Genom Labs Pvt. Ltd, Cochin, India.

#### Random Amplified Polymorphic DNA (RAPD) and Inter Simple Sequence Repeats (ISSR) analysis

For RAPD profiling, eleven random decamer primers (Imperial Life Sciences (P) Ltd, (Table 1) were used to produce fingerprinting profiles. The reactions were assembled in a Veriti Thermal Cycler (Applied Biosystems, Singapore) in a total volume of 50 μl containing 5.0 μl of 10X reaction buffer, 1.0 mM MgCl_2_, 0.5 mMdNTPs, 5 pmols of each primer, 1 unit of Taq DNA polymerase and 50 ng template DNA. Negative control reaction without DNA was included in each set. The PCR amplification was carried out with the following conditions: initial denaturation at 95°C for 5 min, followed by 35 cycles of denaturation at 94°C for 35s, annealing at 32°C for 45 s, extension at 72°C for 2 min and a final extension of 10 min at 72°C. The amplified PCR product was separated on 1.5% agarose gel using TAE buffer and examined under gel documentation system as described earlier. Ten ISSR primers were selected (Table 1) and amplification was done as per Du et al. [33].

**Table 1:** RAPD and ISSR primers and corresponding annealing temperatures used.

Marker	Primer Code	Primer Sequences (5’-3’)	Annealing temperature (°C)
RAPD	R1	ggacttacag	32.0
	R2	ctgactcatg	34.0
	R3	cttcattgcc	36.0
	R4	tgccgagctg	30.0
	R5	aatcgggctg	32.0
	R6	gaaacgggtg	32.0
	R7	gtgacgtagg	34.0
	R8	agccagcgaa	36.0
	R9	caaacgtcgg	36.0
	R10	gtcgccgtca	34.0
	R11	ccgcatctac	34.0
ISSR	P1	tg(ca)_6_c	50.0
	P2	gt(ca)_6_c	50.0
	P3	gtgacga(ct)_6_	52.0
	P10	(ga)_8_c	54.0
	P11	(ag)_8_c	52.0
	P12	(ga)_8_c	52.0
	P13	(ca)_8_g	54.0
	P14	(tc)_8_c	54.0
	P15	(ac)_8_c	54.0
	P16	(ag)_8_g	50.0

The PCR reaction mixture was performed in 25 μl total volume, containing 50 ng of genomic DNA, 2.5 μl of 10X Taq Buffer with 15mM MgCl_2_, 2.0 μl of 2.5 mM dNTPs, 0.5 μl of 10 pmol ISSR primer,1 μl of DMSO (100%), 0.5 μl of BSA (10 mg/ml) and 2U *Taq* DNA Polymerase.The amplification conditions were as follows: initial denaturation at 94°C for 4 min followed by 35 cycles at 94°C for 50 s, at 45°C for 50 s, and at 72°C for 2 min with a final extension step at 72°C for 7 min. The amplified fragments were separated on 1.2% agarose gel using 1X TAE buffer and the banding patterns were examined under gel documentation system asmentioned above.

#### Phylogenetic analysis

The obtained ITS sequences were compared with type strains retrieved from GenBank/NCBI database using BlastN search program and were aligned using Clustal W [34]. Evolutionary models were selected according to BIC scores (Bayesian Information Criterion) and AICc values (Akaike Information Criterion, corrected) [35] using Mega 5.05 version [34]. The phylogenetic tree was constructed by neighbor-joining method using the model Kimura 3-parameter K2 + I (I = 0.67, R = 1.67) [36] taking *Solanumkurzii*(KC535794) as an out group. The robustness of the phylogenetic tree was tested by bootstrap analysis using 1,000 replicates using *p*-distance model [37]. Trees were viewed and edited by using program FigTree 1.3.1.

Amplified fragments of RAPD and ISSR were scored as present (1), or absent (0), the fragments produced were numbered sequentially and entered into a binomial matrix [38]. Only fragments bigger than 100 bases were considered for analysis. The binary matrix was used to calculate the Simple Matching (SM) coefficient, and a cluster analysis was performed using Unweighted Pair Group with Arithmetic Mean (UPGMA) algorithm supported by Numerical Taxonomy SYStem (NT_SYS_ version 2.2).

### Determination of Nutritional values of selected wild edible mushrooms

#### Ash content

Samples (1 g each) were dried for overnight in the oven maintained at 105°C and weighed, then placed at 575°C for 5 h. Then, the samples were weighed for final mass and difference between the mass at 105°C and 575°C was taken as ash content [39].

#### Carbohydrate content

Carbohydrate content was estimated by phenol sulphuric acid method with glucose as standard [40].

#### Protein content

Protein content was estimated by Bradford method using BSA as standard [41].

#### Fat content

The fat was extracted by Chloroform-methanol extraction and estimation method [42].

### Determination of antioxidant potential of selected wild edible mushrooms

#### Extract preparation

The lyophilized powder (1.5 g) was extracted by stirring with 40 mL of methanol (25°C at 150 rpm) for 2 h and subsequently filtered through Whatman No. 4 paper. The residue was then extracted with 20 mL of methanol (25°C at 150 rpm) for 2 h. The combined methanolic extracts were evaporated at 40°C (rotary evaporator BUCHI R-210, Switzerland) to dryness and stored at 4°C until analysis [43].

#### Determination of total phenolic content (TPC)

Total phenolic content was measured according to the method given by Singleton et al. [44]. Briefly, 0.5 mL of sample was added to 4.5 mL of distilled water and was mixed with 0.2 mL saturated solution of Na_2_CO_3;_ finally 4.3 mL of distilled water was added to the solution. The reaction mixture was incubated for 60 min in the dark at room temperature and then the absorbance was measured at 725 nm using a microplate spectrophotometer UV-vis (Multiscan^™^ GO, Thermo Scientific, MA, USA). Total phenolic content was expressed as mg of Gallic acid equivalents (GAE) per gram of dry sample (mg GAE/g). The measurements were done in triplicate.

#### Determination of total flavonoids content (TFC)

The flavonoids content was determined by the calorimetric method of aluminium chloride according to methodology previously described by Chang et al [45]. In this, 0.5 mL was mixed with 1.5 mL of 95% ethanol, 0.1 mL of 10% aluminium chloride, 0.1 mL of 1M potassium acetate and 2.8 mL of distilled water. Finally, absorbance was measure at 415 nm using a microplate spectrophotometer UV-vis (Multiscan^™^ GO, Thermo Scientific, MA, USA) after incubation for 30 min in darkness at room temperature. The flavonoid content was calculated in mg Quercetin equivalents per g of dried sample (mg QAE/g).

#### Scavenging Activity of ABTS Radical

ABTS radical scavenging activity was determined according to Re et al [46] with some modifications. 2, 2-azino-bis (3-ethylbenzothiazoline-6-sulphonic acid) (ABTS) was dis- solved in water to a 7 mM concentration. ABTS radical cation (ABTS^**O+**^) was produced by reacting ABTS stock solution with 2.45 mM potassium persulfate (final concentration) and allowing the mixture to stand in the dark at room temperature for 12–16 h before use. The ABTS^**O+**^solution was diluted with water to an absorbance of 0.70 (±0.02) at 734 nm. The reaction mixture consisted of 0.07 mL of extract and 3 mL of the ABTS radical. After incubation for 6 min, absorbance was determined in spectrophotometer at 734 nm. The antioxidant activity was calculated by using the following equation.

$$\text{\% inhibition}=\left[\frac{{\text{A}}_{\text{control}}-{\text{ A}}_{\text{sample}}}{{\text{A}}_{\text{control}}}\right]\times 100$$

A_control_ = Absorbance of negative control at the moment of solution preparation

A_sample_ = Absorbance of sample after 45 min

The IC_50_ values were calculated as the concentration of the sample (mg) required to scavenge 50% of the ABTS free radical. The IC_50_ is often used to express the amount of concentration of extracts need to scavenge 50%of the free radicals.

#### Fourier Transform infrared Spectroscopy (FT-IR) analysis of selected wild edible mushrooms

The selected wild mushroom samples were dried and mixed with FT-IR grade potassium bromide (1:20; 0.02 of sample with KBr at a final weight of 0.4 g). The samples were grounded in agate pestle and mortar for obtaining pellets by hydraulic press. The absorbance Fourier transform infrared (FT-IR) spectra of the samples were recorded using JASCO FT-IR 6800 within the scanning range of 400–4000 cm^-1^ and 64 scans per second were recorded [47].

### Data analysis

The data were calculated in mean ± SD of the values found in triplicates from antimicrobial screening and analysed by one way of analysis of variance (ANOVA) to compare statistical significant differences (P<0.05) between antimicrobial activities of all isolates using Duncan’s multiple range test of SPSS software version 6.0. All the experiments were done in triplicates and the mean was taken. Relative abundance of mushroom isolates was compared between the selected natural forests by using Sigma Plot 12.0 version. Venn diagram was prepared online by using Bioinformatics and evolutionary genomics site (http://bioinformatics.psb.ugent.be/webtools/Venn/).

### Nucleotide sequence accession numbers

All the ITS rRNA gene sequences were deposited in NCBI-GenBank with accession numbers (KJ865831- KJ865841, KJ865843, KM985651, KM985653- KM985664, and KM985666- KM985685).

## Results

### Assessing Mushroom diversity

**Fig 2:**
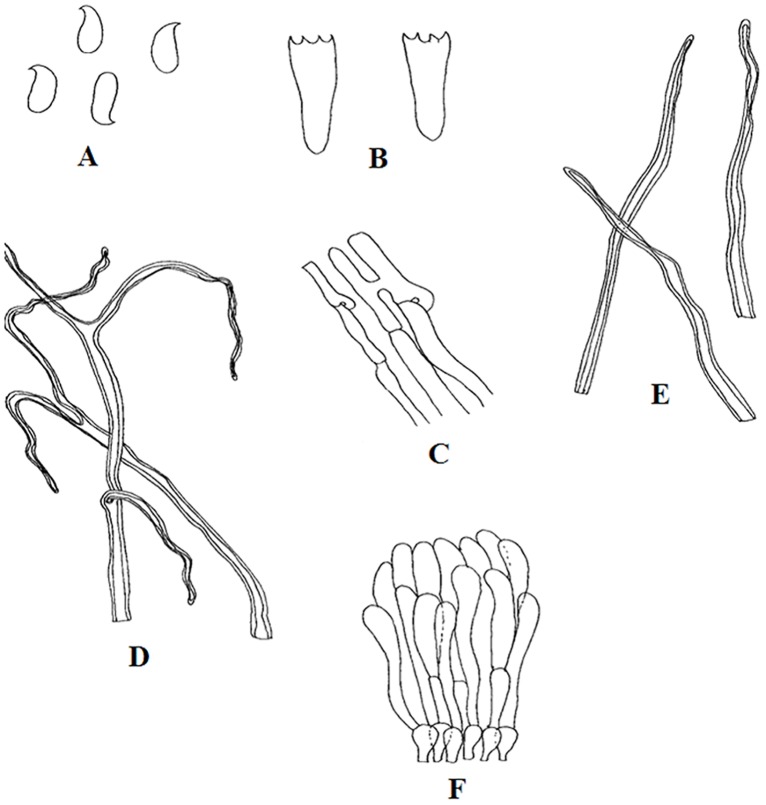
*Lentinus*sp.: A. Basidiospores, B. Basidia, C. Generative hyphae, D. Sketetalhyphae, E. Binding hyphae, F. Hyphal pegs.

**Fig 3:**
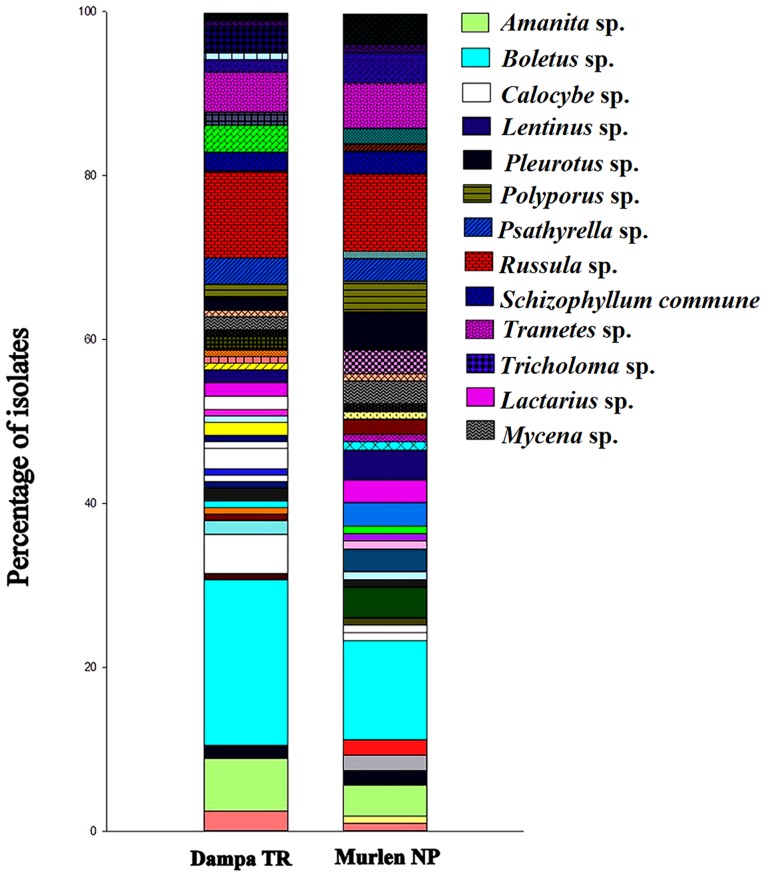
Relative abundance of wild mushrooms at the genus level in Dampa TR and Murlen NP.

**Fig 4:**
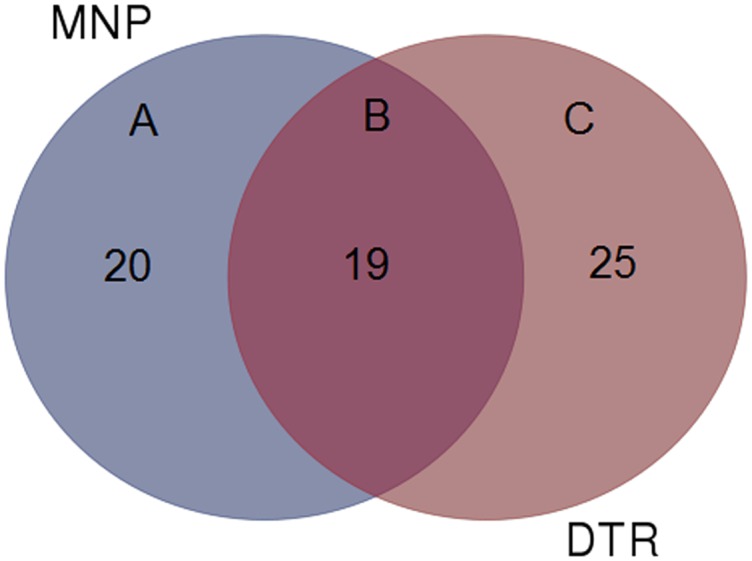
Venn diagram showing the number of genera shared among the two forests (B), present in DTR (C) and MNP (A).

The present study documented 231 wild mushrooms, distributed across 64 genera belonging to 36 families, 14 orders and 06 classes. Morphologically, all isolates were classified into two Phylum Basidiomycota (n = 221, 95.67%) and Ascomycota (n = 10, 4.32%). Anatomical characteristics were also recorded of all collected fruiting bodies (Fig 2). Some of the economically important genus found are *Lactarius*, *Trametes*, *Calocybe*, *Agaricus*, *Pleurotus*, *Schizophyllum*, *Cantharellus*, *Russula*, *Boletus*, *Termitomyces*, *Pholiota* and *Lentinus*. Relative abundance of the wild mushrooms was compared between the two selected forests at the genus level (Fig 3) and the result showed that *Boletus* was the most abundant genus at DTR and MNP with 20.16 and 12.1% respectively. Genus *Russula*(10.48%) and *Amanita* (6.45%) were the most dominant genus in DTR as compared to MNP with 9.34% and 3.73% respectively. At the same time, genus *Mycena* and *Trametes* were dominant in MNP with 2.8% and 5.6% respectively. However, 19 genera were found in common among both the studied locations (Fig 4). Moreover, some rare genus like *Favolaschia* and *Cookeina* were found only in DTR. These results indicated that diversity of wild macrofungi varies between these two places due to different climatic conditions. MNP is known for its dense forest where only 1% sunlight can directly penetrate the forest on a sunny day. It has clayey fine soil which is usually with severe erosion. On the other hand, DTR being on a lower elevation with fine loamy soil, which is richer in humus than the soil type found in MNP. It also has an ideal climatic condition like higher humidity and warmer temperature to support mycelial growth of fungi. All these factorscould be the cause that led the higher diversity of wild mushrooms in DTR than in MNP.

### Antimicrobial activity

Out of 231, 76 isolates could be grown on artificial media and were screened for their antimicrobial potential. All isolates were tested for their *in vitro* antagonistic activity against the selected fungal plant pathogens by dual culture plate assay and showed diverse antagonistic effects. Out of 76, 45 isolates showed positive growth inhibitory activity against atleast three fungal pathogens, with the percentage of inhibition ranging from 25% to 85% (Table 2). All isolates showed positive activity against *Fusarium oxy* f. sp. *ciceri* and *F*. *oxysporum* whereas *C*. *albicans* was the most susceptible pathogen against all wild macrofungi with the exception of 15 isolates BPSM (01, 05, 14, 22, 29, 30, 31, 33, 37, 38, 43, 45, 46, 47 and 48). Five isolates BPSM (31, 33, 37, 38 and 45) exhibited positive antagonistic activity against all the tested pathogens. Among them, isolate BPSM30 indicated highest antifungal activity (78%) against *F*. *proliferatum* whereas isolate BPSM41 showed maximum antifungal activity (80%) against *F*. *oxy* f. sp. *ciceri*. The strongest antagonistic activity was found in BPSM33 isolate against *F*. *oxysporum* with 85.71% inhibition as compared to control.

**Table 2:** Antimicrobial activity of wild mushrooms against selected bacterial and fungal pathogens.

Sl. No.	Antifungal activity (percentage of inhibition ± SD)				Antibacterial activity (zone of inhibition in mm ± SD)		
					*Gram positive*		*Gram negative*
	*F*. *proliferatum*	*F*. *oxy* f. sp. *ciceri*.	*F*. *oxysporum*	*C*. *albicans*	*P*. *aeruginosa*	*E*. *coli*	*S*. *aureus*
BPSM01	30.23±0.14^a^	30.6±0.14^a^	29.05±0.21^a^	6.3±0.2^a^	9.63±0.15^a^	-	9.6±0.1^a^
BPSM02	-	42.85±0.14^b^	35.56±0.07^b^	-	5.5±0.26^bc^	-	6.36±0.11^bc^
BPSM03	44.18±0.14^b^	55.1±0.14^d^	48.89±0.07^c^	-	6.16±0.15^bde^	5.36±0.25^a^	4.36±0.2^bde^
BPSM04	32.55±0.07^a^	36.73±0.07^b^	26.67±0.07^a^	-	5.46±0.2^bc^	-	-
BPSM05	51.16±0.07^c^	46.93±0.14^c^	40.00±0.07^b^	6.26±0.11^a^	9.3±0.2^a^	-	9.26±0.2^a^
BPSM06	41.86±0.07^b^	40.81±0.07^b^	42.23±0.21^b^	-	6.26±0.26^bde^	-	6.26±0.15^bc^
BPSM07	53.48±0.07^c^	55.1±0.14^d^	44.45±0.07^b^	-	5.46±0.2^bc^	-	6.2±0.2^bc^
BPSM08	41.86±0.07^b^	42.85±0.14^b^	46.67±0.21^c^	-	5.93±0.15^bc^	-	6.63±0.2^bc^
BPSM09	-	48.33±0.21^c^	45.71±0.21^c^	-	4.36±0.23^bdfg^	-	4.66±0.2^bde^
BPSM10	30.93±0.21^a^	28.57±0.07^a^	26.23±0.07^a^	-	9.33±0.2^a^	-	8.26±0.15^bdfg^
BPSM11	30.23±0.07^a^	36.73±0.07^b^	42.23±0.07^b^	-	4.3±0.26^bdfg^	-	6.33±0.15^bcfhi^
BPSM13	28.05±0.14^a^	28.36±0.28^a^	25.56±0.14^a^	-	6.3±0.2^bde^	5.23±0.12^a^	5.63±0.15^bdfhjk^
BPSM14	39.53±0.07^b^	30.2±0.28^a^	-	7.9±0.05^bc^	5.26±0.15^bc^	-	4.43±0.2^bde^
BPSM16	48.83±0.14^c^	53.06±0.07^c^	55.56±0.14^d^	-	6.26±0.05^bde^	-	4.36±0.2^bde^
BPSM17	33.95±0.07^a^	32.24±0.07^a^	34.45±0.07^a^	-	-	-	5.63±0.11^bdfhjk^
BPSM18	-	32.24±0.07^a^	25.56±0.14^a^	-	5.36±0.2^bc^	-	5.7±0.26^bdfhjk^
BPSM19	34.88±0.14^a^	30.61±0.14^a^	33.34±0.14^a^	-	6.26±0.2^bde^	-	4.16±0.2^bde^
BPSM20	-	48.97±0.21^c^	44.45±0.07^b^	-	6.96±0.15^bdfhi^	-	4.26±0.15^bde^
BPSM21	-	30.4±0.07^a^	34.45±0.21^a^	-	5.23±0.15^bc^	-	5.66±0.11^bdfhjk^
BPSM22	29.3±0.07^a^	28.57±0.21^a^	26.67±0.07^a^	7.86±0.11^bc^	5.23±0.2^bc^	-	6.33±0.2^bc^
BPSM23	-	34.28±0.14^a^	28.89±0.07^a^	-	5.6±0.26^bc^	-	5.33±0.11^bdfjk^
BPSM24	28.6±0.07^a^	26.48±0.07^a^	26.67±0.07^a^	-	6.06±0.15^bde^	-	-
BPSM25	33.25±0.07^a^	51.02±0.14^c^	30.00±0.14^a^	-	6.13±0.2^bde^	-	-
BPSM26	33.95±0.07^a^	30.61±0.07^a^	35.56±0.21^b^	-	6.1±0.2^bde^	-	-
BPSM27	32.85±0.28^a^	33.8±0.14^a^	37.49±0.07^b^	-	8.16±0.2^bdfhj^	-	4.76±0.11^bde^
BPSM29	56.67±0.21^d^	52.38±0.21^c^	42.85±0.14^b^	4.66±0.15^bde^	5.43±0.15^bc^	-	-
BPSM30	78.33±0.21^f^	74.6±0.14^e^	71.42±0.28^e^	7.96±0.05^bc^	9.16±0.15^a^	-	8.46±0.11^bdfg^
BPSM31	61.67±0.21^d^	61.9±0.07^d^	53.56±0.28^c^	7.86±0.05^bc^	5.63±0.15^bc^	5.16±0.11^a^	6.23±0.11^bc^
BPSM32	45.03±0.28^c^	41.44±0.07^b^	44.77±0.21^b^	-	-	-	5.5±0.2^bdfhjk^
BPSM33	75.00±0.35^f^	73.01±0.07^e^	85.71±0.14^g^	7.83±0.05^bc^	9.16±0.2^a^	4.33±0.15^b^	8.6±0.26^bdfg^
BPSM34	68.33±0.07^e^	76.18±0.21^f^	69.63±0.07^e^	-	9.16±0.2^a^	-	7.5±0.1^bdfhjl^
BPSM35	55.00±0.07^d^	53.97±0.07^c^	49.99±0.14^c^	-	8.26±0.15^bdfhj^	4.43±0.20^b^	7.06±0.15^bdfhjl^
BPSM36	63.33±0.14^d^	55.55±0.56^d^	66.06±0.07^e^	-	9.43±0.2^a^	-	7.73±0.2^bdfhjl^
BPSM37	55.00±0.07^d^	76.18±0.35^f^	51.78±0.07^c^	4.23±0.0^5bde^	8.2±0.26^bdfhj^	4.23±0.11^b^	7.3±0.17^bdfhjl^
BPSM38	61.66±0.07^d^	44.44±0.07^b^	42.85±0.28^b^	7.96±0.05^bc^	6.63±0.2^bde^	5.13±0.15^a^	5.73±0.15^bdfhjk^
BPSM39	44.85±0.14^b^	45.04±0.14^c^	42.06±0.07^b^	-	5.63±0.2^bc^	-	-
BPSM40	-	39.28±0.14^a^	37.49±0.07^b^	-	7.06±0.15^bdfhi^	-	4.5±0.26^bde^
BPSM41	76.66±0.14^f^	80.95±0.14^f^	62.49±0.21^d^	-	9.43±0.05^a^	-	7.6±0.1^bdfhjl^
BPSM42	26.67±0.07^a^	28.23±0.07^a^	25.44±0.14^a^	-	6.23±0.15^bde^	-	-
BPSM43	56.66±0.14^d^	55.55±0.14^d^	66.06±0.07^e^	4.53±0.05^bde^	5.33±0.2^bc^	-	-
BPSM44	-	34.56±0.07^a^	37.28±0.14^b^	-	5.73±0.15^bc^	-	5.66±0.15^bdfhjk^
BPSM45	56.66±0.28^d^	55.56±0.14^d^	55.3±0.35^d^	4.36±0.05^bde^	7.26±0.2^bdfhi^	4.93±0.15^a^	7.56±0.2^bdfhjl^
BPSM46	56.67±0.07^d^	58.72±0.28^d^	49.99±0.14^c^	6.36±0.15^a^	9.46±0.2^a^	-	9.33±0.11^a^
BPSM47	55.00±0.21^d^	49.2±0.14^c^	51.78±0.21^c^	6.36±0.11^a^	9.56±0.25^a^	-	9.46±0.2^a^
BPSM48	48.33±0.07^c^	47.61±0.21^c^	49.99±0.14^c^	6.36±0.05^a^	9.6±0.26^a^	-	9.26±0.15^a^

Mean (±SD) followed by the same letter(s) in each column are not significantly different at P <0.5 using Duncan’s new multiple range test.

All isolates were tested for their antibacterial activity against three bacterial pathogens viz. *P*. *aeruginosa*, *S*. *aureus* and *E*. *coli*. Out of 76 isolates, 37 isolates showed positive effects against two out of three pathogens and all isolates showed positive effect against *P*. *aeruginosa* except isolates BPSM17 and BPSM32. *E*.*coli*is the strongest pathogen against all the tested isolates except BPSM (03, 13, 31, 33, 37, 38, 45 and 55). The result showed that isolate BPSM01 displayed greatest inhibitory effect (9.63 mm) followed by BPSM48 (9.6 mm) and BPSM47 (9.56 mm) against *P*. *aeruginosa*. The result indicated that isolates BPSM01 and BPSM47 had the most significant effect against *S*. *aureus*growth with 9.6 mm and 9.46 mm respectively. Isolate BPSM03 exhibited broad spectrum antibacterial activity against *E*. *coli*(5.36 mm) and *S*. *aureus* (4.36 mm), therefore it can be considered as the most potent isolate for further studies (Table 2).

### ITS rRNA gene amplification and phylogenetic analysis

**Fig 5:**
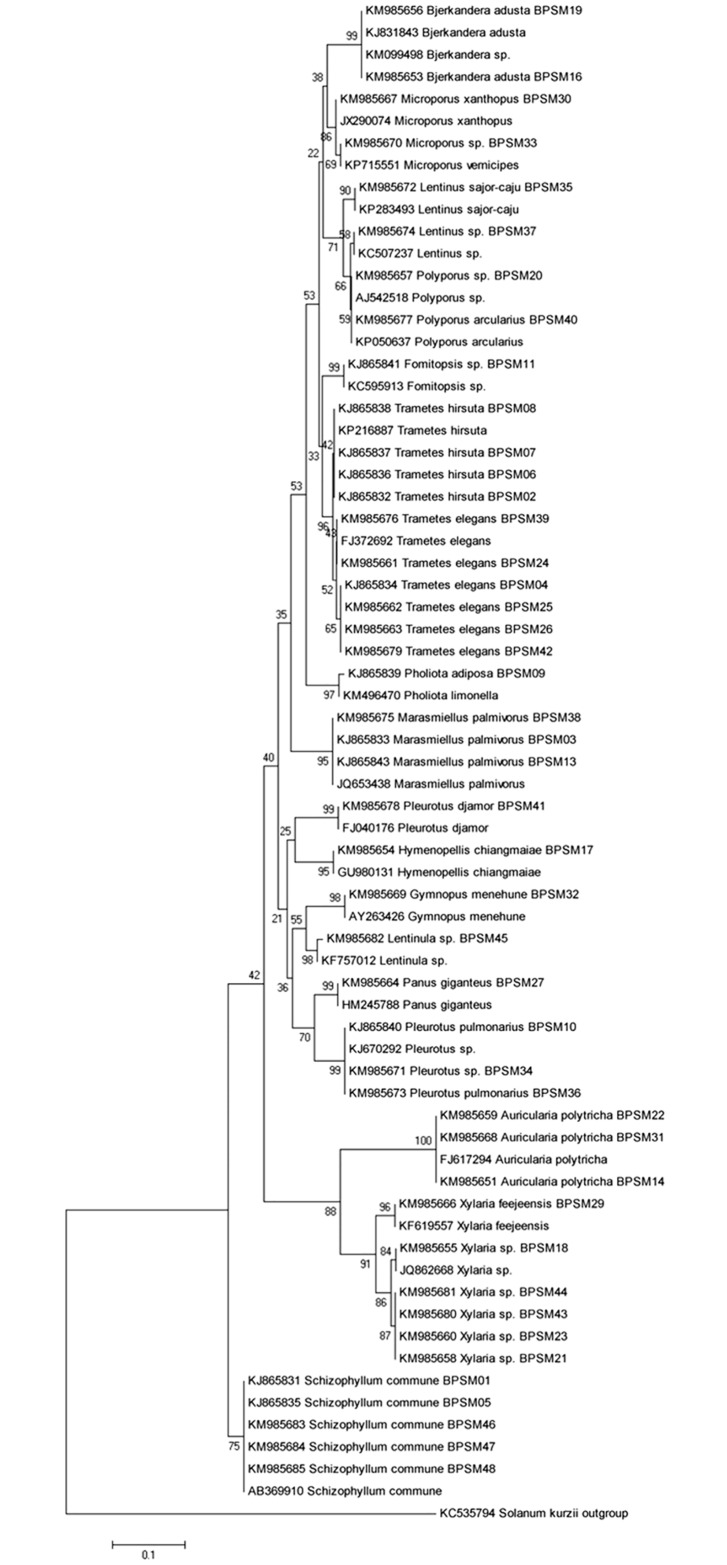
Neighbor-joining phylogenetic treeusing Kimura 2-parameter model based on ITS gene sequence of wild edible mushrooms. Numbers at branches indicate bootstrap values of neighborjoining analysis (>50%) from 1,000 replicates.

Forty-five isolates which showed significant antimicrobial potential against tested bacterial and fungal pathogens were subjected for the amplification of ITS rRNA gene. The obtained sequences were analyzed using the BLAST analysis tool. All isolates showed 97–100% similarity with the sequence available in NCBI database (Table 3). Most of the isolates belongs to genus *Trametes* (n = 8, 17.77%) followed by *Xylaria* (n = 6, 13.33%), *Schizophyllum* (n = 5, 11.11%), *Pleurotus* (n = 4, 8.88%), *Marasmiellus* (n = 3, 6.66%), *Microporus* (n = 3, 6.66%), *Auricularia* (n = 3, 6.66%), *Polyporus* (n = 3, 6.66%), *Lentinus* (n = 2, 4.44%), *Bjerkandera* (n = 2, 4.44%), *Panus* (n = 1, 2.22%), *Gymnopus* (n = 1, 2.22%), *Pholiota* (n = 1, 2.22%), *Fomitopsis* (n = 1, 2.22%), *Hymenopellis* (n = 1, 2.22%) and *Lentinula* (n = 1, 2.22%). The phylogenetic tree was constructed based on the neighbor-joining method with Kimura 2-parameter model in accordance with lowest BIC and highest AICc values using Mega 5.05. Transition/Transversion values were also estimated using Mega 5.05 (R = 1.75). ITS rRNA gene analysis classified the isolates into 16 genera and 11 families (Table 4). The phylogenetic tree showed that isolates were divided into two separate clades with bootstrap supported value of 65% and 75% respectively. Clade I was the largest cluster consisting 40 isolates and divided into two clusters as clade IA and clade IB. Most of the strain the genera *Auricularia* and *Xylaria*, falls in the clade IB group with bootstrap support of 88%. Clade II contains all five isolates identified as *Schizophyllum commune* strains BPSM (01, 05, 46, 47 and 48) with a bootstrap value of 75% (Fig 5).

**Table 3:** Identification of antagonistic wild mushrooms based on ITS rRNA gene sequences.

Isolate No.	Accession number	Closest species with accession number	Similarity	Identification
BPSM01	KJ865831	*Schizophyllum* sp. (KR155096)	99%	*Schizophyllum commune*
BPSM02	KJ865832	*Trametes hirsuta* (KP216914)	99%	*Trametes hirsuta*
BPSM03	KJ865833	*Marasmiellus palmivorus* (JQ653438)	99%	*Marasmiellus palmivorus*
BPSM04	KJ865834	*Trametes* sp. (KP686448)	99%	*Trametes elegans*
BPSM05	KJ865835	*Schizophyllum commune* (AB470852)	99%	*Schizophyllum commune*
BPSM06	KJ865836	*Trametes hirsuta*(JN048768)	99%	*Trametes hirsuta*
BPSM07	KJ865837	*Trametes hirsuta*(KC461301)	99%	*Trametes hirsuta*
BPSM08	KJ865838	*Trametes hirsuta* (KP216887)	99%	*Trametes hirsuta*
BPSM09	KJ865839	*Pholiotalimonella*(KM496470)	98%	*Pholiotaadiposa*
BPSM10	KJ865840	*Pleurotus* sp.(KJ670292)	99%	*Pleurotus pulmonarius*
BPSM11	KJ865841	*Fomitopsis*sp. (KC595913)	99%	*Fomitopsis*sp.
BPSM13	KJ865843	*Marasmiellus palmivorus* (JQ653437)	99%	*Marasmiellus palmivorus*
BPSM14	KM985651	*Auriculariapolytricha* (FJ617294)	100%	*Auriculariapolytricha*
BPSM16	KM985653	*Bjerkandera*sp. (KM099498)	100%	*Bjerkanderaadusta*
BPSM17	KM985654	*Hymenopellischiangmaiae* (GU980131)	100%	*Hymenopellischiangmaiae*
BPSM18	KM985655	*Xylaria*sp. (JQ862668)	100%	*Xylaria*sp.
BPSM19	KM985656	*Bjerkanderaadusta* (KJ831843)	100%	*Bjerkanderaadusta*
BPSM20	KM985657	*Polyporus*sp. (AJ542518)	100%	*Polyporus*sp.
BPSM21	KM985658	*Xylaria*sp. (KP263113)	99%	*Xylaria*sp.
BPSM22	KM985659	*Auriculariapolytricha* (FJ617295)	99%	*Auriculariapolytricha*
BPSM23	KM985660	*Xylaria*sp. (KM066560)	97%	*Xylaria*sp.
BPSM24	KM985661	*Trametes elegans* (JN048766)	100%	*Trametes elegans*
BPSM25	KM985662	*Trametes* sp. (FJ372692)	99%	*Trametes elegans*
BPSM26	KM985663	*Trametes elegans* (JN164936)	99%	*Trametes elegans*
BPSM27	KM985664	*Pleurotus* sp. (HQ668461)	99%	*Panusgiganteus*
BPSM29	KM985666	*Xylariafeejensis* (KF619557)	100%	*Xylariafeejensis*
BPSM30	KM985667	*Microporusxanthopus* (JX290074)	99%	*Microporusxanthopus*
BPSM31	KM985668	*Auriculariapolytricha* (FJ617294)	100%	*Auriculariapolytricha*
BPSM32	KM985669	*Gymnopusmenehune* (AY263426)	99%	*Gymnopusmenehune*
BPSM33	KM985670	*Microporusvernicipes* (KP715551)	100%	*Microporus* sp.
BPSM34	KM985671	*Pleurotus pulmonarius* (KF932728)	99%	*Pleurotus* sp.
BPSM35	KM985672	*Lentinussajor-caju* (KP283493)	99%	*Lentinussajor-caju*
BPSM36	KM985673	*Pleurotus pulmonarius* (FJ379269)	100%	*Pleurotus pulmonarius*
BPSM37	KM985674	*Lentinus*sp. (KC507237)	99%	*Lentinus*sp.
BPSM38	KM985675	*Marasmiellus palmivorus*(JQ653433)	99%	*Marasmiellus palmivorus*
BPSM39	KM985676	*Trameteselegans*(KF573029)	99%	*Trametes elegans*
BPSM40	KM985677	*Polyporusarcularius* (KP050637)	100%	*Polyporusarcularius*
BPSM41	KM985678	*Pleurotusdjamor* (FJ040176)	100%	*Pleurotusdjamor*
BPSM42	KM985679	*Trametes elegans* (JN164921)	99%	*Trametes elegans*
BPSM43	KM985680	*Xylaria*sp. (JN615250)	97%	*Xylaria*sp.
BPSM44	KM985681	*Xylaria*sp. (JX082389)	97%	*Xylaria*sp.
BPSM45	KM985682	*Lentinula* sp. (KF757012)	99%	*Lentinula* sp.
BPSM46	KM985683	*Schizophyllum commune* (AB369910)	100%	*Schizophyllum commune*
BPSM47	KM985684	*Schizophyllum commune* (KP326577)	99%	*Schizophyllum commune*
BPSM48	KM985685	*Schizophyllum commune* (JX848644)	99%	*Schizophyllum commune*

**Table 4:** ITS rRNA gene analysis classified the isolates into 16 genera and 11 families.

Sl.No.	Genus	No. of individuals	%	Family
**1**	*Auricularia*	3	6.67	Auriculariaceae
**2**	*Fomitopsis*	1	2.22	Fomitopsidaceae
**3**	*Schizophyllum*	5	11.11	Schizophyllaceae
**4**	*Gymnopus*	1	2.22	MarasmiaceaeMarasmiaceaeMarasmiaceae
**5**	*Marasmiellus*	3	6.67	
**6**	*Lentinula*	1	2.22	
**7**	*Bjerkandera*	2	4.44	Meruliaceae
**8**	*Hymenopellis*	1	2.22	Physalacriaceae
**9**	*Pleurotus*	4	8.88	Pleurotaceae
**10**	*Panus*	1	2.22	PolyporaceaePolyporaceaePolyporaceaePolyporaceaePolyporaceae
**11**	*Trametes*	10	22.22	
**12**	*Microporus*	2	4.44	
**13**	*Lentinus*	2	4.44	
**14**	*Polyporus*	2	4.44	
**15**	*Pholiota*	1	2.22	Strophariaceae
**16**	*Xylaria*	6	13.33	Xylariaceae

### RAPD analysis

**Fig 6:**
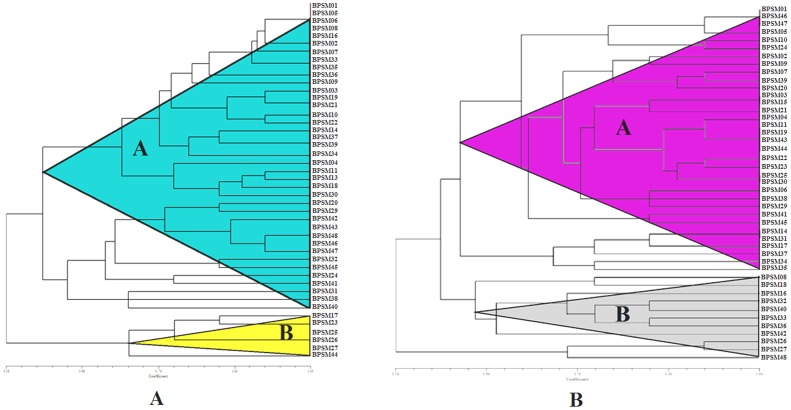
UPGMA dendrogram generated from (A) RAPD and (B) ISSR markers of 45 wild mushrooms using Ntsys 2.0.

RAPD analysis was employed to detect genotypic variation among 45 selected strains using eleven random primers, which yielded 337 fragments. Amplified fragments in each strain were ranging from four to twelve bands which varied in size from 200 bp to 2500 bp. Of the 337 amplified bands, 272 were polymorphic with an average of 8.4 polymorphic fragments per primer. The percentage of minimum polymorphic ranging from 34.2% (R1) to maximum polymorphic of 78.8% (R5) was observed with an average of 56.8% polymorphism. Two primers showed more than 46% polymorphism. The dendrogram based on RAPD data was constructed by UPGMA analysis. The dendrogram grouped the 45 isolates into two clusters (A & B) with similarity coefficient ranges from 0.58 to 1.00. Cluster A was the largest cluster comprised 39 isolates and divided into two sub clusters A1 and A2. Cluster A1 consist of 25 isolates i.e. *Xylaria*sp., *Trametes* sp., *Microporus* sp., *Lentinus*sp., *Pleurotus* sp., *Pholiota*sp., *Marasmiellus* sp., *Bjerkandera*sp., *Auricularia*sp., *Fomitopsis*sp. and Cluster A2 contain 14 isolates i.e. *Pleurotus* sp., *Schizophyllum* sp., *Gymnopus*sp., *Lentinula* sp., *Trametes* sp., *Lentinus*sp., *Marasmiellus* sp., *Polyporus*sp. Six isolates of *Trametes* sp. were clustered together and formed cluster B. In clade B, *Schizophyllum* sp. was clustered with *Pleurotus* sp. and they were very closely similar (Fig 6A).

### ISSR analysis

All the potential isolates were further screened to generate ISSR polymorphic DNA banding pattern using ten primers which varied in size from 200 bp to 2500 bp. Of the 312 amplified bands, 259 were polymorphic with an average of 10.4 per primer. Maximum percentage of polymorphic was observed using primer P1 (92.4%) while the minimum percentage of polymorphic was observed using primer P2 (38.6%) with an average of 72.5%. Two primers showed more than 52% polymorphism. Dendrogram was constructed by UPGMA on the basis of the distance matrix by ISSR analysis. It divided the isolates into two clusters (A & B) with similarity coefficient ranges from 0.59 to 1.00. Cluster A was the largest cluster containing 42 isolates. Cluster A divided into two sub cluster A1 and A2. Cluster A1 consist of 34 isolates and Cluster A2 contain 8 isolates. Cluster B was composed of 3 isolates all belong to genus *Trametes*. Though, the dendrogram was similar with RAPD analysis, there was a slight difference from RAPD analysis. For example, *Schizophyllum* sp. BPSM (01, 05, 46, 47, 48) had the closest relationship with *Pleurotus* sp. BPSM (10, 34) in the RAPD analysis, while it had similarly found in ISSR analysis where *Schizophyllum* sp. had the closest relationship with *Pleurotus* sp. Cluster B showed all the isolates belongs to *Trametes* in the RAPD analysis, while cluster B showed highly similar result in ISSR analysis (Fig 6B).

### Nutritional values of selected wild edible mushrooms

Nutritional value and energetic contribution of the selected six wild mushrooms is presented in Table 5. *Pleurotusdjamor* BPSM41 (21.4 g/100g dry weight, dw) showed the highest level of ash content among the mushrooms studied. Three out of six mushrooms (*S*. *commune* BPSM01, *Pleurotus* sp. BPSM34 and *Panusgiganteus*BPSM27) showed almost the same level of proteins 30.3, 34.3 and 35.59 g/100 dw respectively. Carbohydrates content is highest in *Panusgiganteus*BPSM27 (80 g/100g dw), on the other hand the level of carbohydrates is extremely lower in the other samples ranging from 20–80 g/100g dw. Regarding fat contents, *Lentinula* sp. BPSM45 showed the highest fat content (6.26 g/100g dw) and *Pleurotus*sp. BPSM34 revealed the lowest fat content (2.32 g/100g dw).

**Table 5:** Determination of Ash, Protein, Carbohydrate and Fat content in selected wild mushrooms.

SN	Mushroom Samples	Ash (g/100g)	Proteins (g/100g)	Carbohydrate (g/100g)	Fat content (g/100g)
1	*Schizophyllum commune* BPSM01	6.317	30.33	30.01	5.25
2	*Pleurotus* sp. BPSM34	8.866	34.30	40.05	2.32
3	*Panusgiganteus* BPSM27	7.749	35.59	80.61	2.59
4	*Lentinus*sp. BPSM37	5.257	24.05	20.33	2.72
5	*Lentinula* sp. BPSM45	21.406	16.90	40.15	6.26
6	*Pleurotusdjamor* BPSM41	8.443	12.21	30.28	3.31

### Antioxidant potential

Bioactive properties of the selected six edible mushrooms were assessed by three different assays measuring total phenolic contents, total flavonoids content and scavenging activity of ABTS radical (Table 6). As shown in the table, the total phenol content (TPC) of all the tested samples was ranging from 12.76 to 55.19 mg GAE/g (Gallic Acid Equivalents per gram of dried sample).

**Table 6:** Antioxidant activity of wild mushrooms.

SN	Scientific name with Sample ID	TPC (μg GAE/mg)	TFC (μg QAE/mg)	ABTS IC_50_ (mg/ml)
1	*Schizophyllum commune* BPSM01	55.19±1.42	0.410±0.005	3.25±0.97
2	*Pleurotus* sp. BPSM34	41.44±0.81	0.528±0.003	5.59±0.7
3	*Panusgiganteus* BPSM27	17.53±1.32	0.373±0.002	11.31±0.84
4	*Lentinus*sp.BPSM37	12.76±0.6	0.382±0.001	11.43±0.82
5	*Lentinula* sp.BPSM45	18.09±0.71	0.652±0.002	5.52±0.6
6	*Pleurotusdjamor* BPSM41	52.27±0.44	0.744±0.003	3.73±1.08

*S*. *commune* BPSM01 exhibited the highest TPC value among the studied mushrooms (55.19 mg GAE/g) followed by *Pleurotusdjamor*(BPSM41) and *Pleurotus*sp. (BPSM34) with 52.27 and 41.44 mg GAE/g, respectively. The lowest TPC value was recorded in *Lentinus*sp. (BPSM37) with 12.76 mg GAE/g. Total flavonoid content (TFC) was quantified as milli-gram of Quercetin equivalent per gram of extract ranges from 0.373 to 0.744 mg of QAE/g. *P*. *djamor* BPSM41 showed the maximum TFC (0.744 mg QAE/g) which is followed by *Lentinula* sp. BPSM45 and *Pleurotus* sp. BPSM34 containing 0.652 mg QAE/g and 0.528 mg QAE/g, respectively. *Panusgiganteus* BPSM27 (0.373 mg QAE/g) and *Lentinus*sp. BPSM37 (0.382 mg QAE/g) showed the lowest values of TFC among the mushrooms studied.

In this study for scavenging activity of ABTS radical, the IC_50_ values range from 3.25 to 11.43 mg/ml. The lower the IC_50_ value of mushroom extract, the higher was their free radical scavenging activity. Among the mushroom extracts we have studied, *S*. *commune* BPSM01 with an IC_50_ of 3.25 mg/ml and *Pleurotusdjamor* BPSM41 with an IC_50_ of 3.73 mg/ml indicates a significant antioxidant activity in the methanolic extract of these mushrooms.

### FTIR Analysis

Mushroom samples were analysed in ATR-FT-IR (Jasco, Japan) and different wave numbers, functional groups and their corresponding polymer of mushroom sample were presented in Table 7. From the results it was evident that, mushroom samples showed perfect peaks for cellulose, hemicelluloses and lignin at the corresponding wave numbers. Hemicellulose peak intensity of wave number 1740 cm^-1^ with functional group of ketone/aldehyde C = O stretch was greatly reduced in *P*. *giganteus* BPSM27 when compared to *S*. *commune* BPSM01, it shows that solubilization of the hemicellulose has taken place in *P*. *giganteus* BPSM27. The hemicellulose peak is totally absent in *Pleurotus* sp. BPSM34, *Lentinus*sp. BPSM37 and *Lentinula* sp. BPSM45; it might be due to the complete solubilization of the hemicellulose part present in the samples. Reduction in lignin peak intensity of *S*. *commune* BPSM01 of wave number 2937 cm^-1^ with a functional group of C-H stretching was observed when compared to sample the rest of the studied mushrooms. Similarly reduction in peak intensity for lignin of wave number 1639 cm^-1^ and 1556 cm^-1^ was noticed in *S*. *commune* BPSM01 and *Lentinus*sp. BPSM37 when compared to *P*. *giganteus* BPSM27, *Pleurotus*sp. BPSM34, *P*. *djamor* BPSM41 and *Lentinula* sp. BPSM45. Functional groups representing cellulose and hemicellulose were observed at wave numbers 1200 cm^-1^ for *P*. *djamor* BPSM41 and *Lentinula* sp. BPSM45. Similarly for *S*. *commune* BPSM01, cellulose and hemicellulose peak with different functional group of glycosidic linkage was noticed at the wave number of 930 cm^-1^. Reduction in peak intensity for *Lentinus*sp. BPSM37 and increase in *P*. *djamor* BPSM41 of wave number 1035 cm-^1^ with a functional group of C-O, C = C, and C-C-O stretching representing cellulose, hemicellulose and lignin was observed respectively, when compared to *S*. *commune* BPSM01, *P*. *giganteus* BPSM27, *Pleurotus*sp. BPSM34 and *Lentinus*sp. BPSM37.

**Table 7:** Assignment of functional group and their corresponding polymer in wild mushrooms.

Wave Number (cm^-1^)	Assignment/Functional Group	Polymer
930	Glycosidic linkage	Cellulose, hemicellulose
1035	C-O, C = C, and C-C-O stretching	Cellulose, hemicellulose, lignin
1200	O-H bending	Cellulose, hemicellulose
1556	Aromatic ring vibration	Lignin
1639	C = O stretching (unconjugated)	Lignin
1740	Ketone/aldehyde C = O stretch	Hemicellulose
2937	C-H stretching	Lignin

## Discussion

More than 200 different mushroom species have been identified from different parts of the world. Previous researchers have shown the importance of mushrooms for the production of novel secondary metabolites having antimicrobial potential [48, 49]. In India, several mushrooms had been reported for their medicinal properties like high antioxidant, antimicrobial and antitumor activities [49, 50]. Few of them are *Agaricusbisporus*[51], *Volvariellabombycina* [52], *Pleurotussajor-caju* [53], *Pleurotuspulmonarius* [54], *Lycoperdonperlatum* [55], *Lentinus tuber-regium* [56] and *Lentinussquarrosulus* [57]. Recently, Metuku et al. [58] reported the application of *Schizophyllumradiatum*in the extracellular biosynthesis of silver nanoparticles and showed antimicrobial activity against gram positive and gram negative bacteria with anti-oxidative properties under submerged fermentation. Moreover, Ramberg et al. [59] suggested that genus *Trametes*has immunomodulatory and anticancer effects.

Among the studied locations, it was revealed that the diversity of wild macrofungi was higher in DTR than in MNP. It is likely that the variation of wild macrofungi in different ecosystems is governed by different climatic conditions such as temperature, pH of soil, physiognomy, succession and geography. This finding was similarly reported by Angelini et al. [60], where sampling methods, soil and microclimate conditions affects diversity of macrofungal species. In addition, level of effectiveness amongst biota and the level of human disturbances may have resulted in difference in species recorded in our study. In our study, we found that diversity of wild macrofungi was higher in the rainy season as compared to the dry season. This finding was similarly reported by Andrew et al. [61] who stated that species diversity was higher in the rainy seasons. Chang and Miles [62] suggested that temperature may be the major physical factor which may affect mycelial growth in mushroom production.

All the wild macrofungi tested were found to have various degrees of antimicrobial effects against gram positive and gram negative bacterial pathogens. Similarly, a previous study has also reported antimicrobial activity from the extract of mushrooms [7]. In our study, the maximum inhibition zone was shown by *Pleurotus pulmonarius* (BPSM36) showing antibacterial activities against *P*. *aeruginosa* (9.4 mm) and *S*. *aureus* (7.7 mm). These findings were similarly reported by Chowdhury et al. [7] who stated that *P*. *ostreatus* has broad spectrum antibacterial activity. Isolate BPSM45 identified as *Lentinula* sp. showed antibacterial activity against all tested bacterial pathogens which was in concordance with the findings of Ishikawa et al. [63] who showed that *Lentinula edodes* has antibacterial action against *B*. *cereus*, *S*. *aureus* and *E*. *coli*. We found that all isolates were highly resistant against *E*. *coli* and *C*. *albicans*. Previous researchers have similarly reported that both the pathogens were found to be strongly effective against mushrooms [7, 55]. Four strains showed significant antimicrobial activity against all the tested bacterial pathogens. Similar antibacterial results were reported by Barros et al. [64], Ramesh and Pattar [55], Chowdhury et al. [7] and Smolskaite et al., [11] who proved that mushrooms has strong antimicrobial activity and are potential candidates for the discovery of antimicrobial products.

All the isolates were screened for their *in vitro* antagonistic activity against three fungal pathogens and 45 isolates (19.48%) showed strong antimicrobial activity against at least two out of three tested pathogens. Interestingly, five isolates (BPSM 31, 33, 37, 38 and 45) found to inhibit all selected fungal pathogens, similar findings were reported by Jagadish et al. [51] and Udu-Ibiam et al. [65] who demonstrated that medicinal mushrooms had found to have antagonistic potential. Isolate BPSM45 identified as *Lentinula* sp.Exhibited the highest percentage of inhibition (55.3%) against *F*. *oxysporum*, which is significantly higher than the finding reported by Osman et al. [66] who showed 22% and 17% inhibition of *F*. *oxysporum*by *Lentinula edodes* LC202 and *Lentinula edodes* LC2141. Most of the selected isolates in the present study showed antagonistic potential against *F*. *oxysporum* f. sp. *ciceri*, a potent pathogen causing diseases in several crops. The potential isolates (BPSM 31, 33, 37, 38 and 45) have shown strong antifungal activity which has the potential to develop as a biocontrol agent. Park et al. [67] has used ITS1 (5’-TCCGTAGGTGAACCTGCGG-3’) and ITS4 (5’-TCCTCCGCTTATTGATAGC-3’) primers to amplify the ITS region to identify isolates of medicinal mushroom *Hericium erinaceum* whereas Dentinger et al. [15] have used ITS sequences to compare with COI and they reported that the overall performances of ITS and COI as barcode markers are similar. However, the barcode locus that has been chosen for animals, COI, will not work for mushrooms, rusts [68], and probably most other fungi [69], primarily due to the variable and unpredictable presence of large introns in the barcode region. The widely used ITS regions works well for identifying species [70,71] and shows that there is no better single molecular for barcoding mushroom species till today, and its versatility makes it possible to survey and discover new fungi. Subsequently, the Internal Transcribed Spacer (ITS) region of the ribosomal DNA (rDNA) has been extensively used as the de facto standard ‘barcoding’ marker for many years [71]. In accordance with the previous reports, we have used ITS region for the identification of selected wild mushrooms.

The analysis of genetic diversity is important to understand the genetic variation among the closely related organisms based on their genetic makeup. In RAPD analysis, both *Pleurotus* sp. (BPSM10 and BPSM34) has been closely clustered together. This finding was similarly reported by Yin et al. [20] and Ro et al.[72] who reported that phenotypic characteristics of fruiting bodies of. *P*. *eryngii* clustered together based on RAPD grouping. It proved that effectiveness of the molecular marker in the genetic diversity analysis of mushrooms at the genus level. The results obtained in the present study provided information regarding the genetic diversity of the strains by combined RAPD/ISSR analysis. In the present study, both RAPD/ISSR fingerprinting analysis clearly showed closely related *Schizophyllum* sp. strains and formed a mono-subclade at the coefficient of 90% and 95% respectively. Similarly *Auriculariapolytricha* (Isolates BPSM14, 22 and 31) clustered together in both RAPD and ISSR technique, which further proved that the selected markers can be used to differentiate at the genus level. The RAPD technique has been successively used to differentiate among different strains of *Agaricus bisporus* [73], *Ganoderma lucidum* complex [74], *Lentinula edodes*[75]; *Agaricusbisporus*[76]. RAPD has the potential to survey the entire genomes, and often identifies multiple loci in a single reaction. RAPD can provide a practical tool for breeders and spawn companies to protect novel strains [77]. *Trametes* strains were clustered together and showed high levels of genetic diversity in ISSR markers, and been used in the identification and taxonomy for more than two decades [78]. It is a very systematic, perceptive and successful method to studygenetic diversity under species level which had been used in *Lentinula edodes*[79], *Cordycepssinensis*[80], *Auriculariapolytricha*[81] and *Agaricus bisporus*[82].

Among the nutritional contribution of the selected six mushrooms we have studied, *Lentinula sp*. BPSM45 showed the highest ash content 21.406 g/100g. On the other hand, our study revealed the highest energetic contribution of *Panusgiganteus* BPSM27 among the selected mushrooms, due to the highest content of carbohydrates and proteins with low fat content. The level of antioxidant activity in mushrooms is largely varied from one species to another and the solvent used for extraction. This could be due to their capability to extract bioactive substances [83]. Apart from these two factors, geographical locations may also affect the bioactive properties of wild mushrooms which could be due to their differences in relation with growth conditions, genetic factors, geographical variations and analytical procedures [84,85,86].

Phenolic group is the major antioxidant compound found in mushrooms, [87]. There are also reducing agents like ascorbic acid and flavonoids contributing in lower values [88, 89]. Phenolic components are potential antioxidants which can donate hydrogen to free radicals to terminate the chain reaction of lipid oxidation at the initial stage. This is due to the phenolic hydroxyl groups which help the phenolic compounds to scavenge radicals [83]. Wong et al [88] reported in methanolic extract of *S*. *commune* 25.03 mg GAE/g where as the TPC of *S*. *commune* BPSM01 was 55.19 mg GAE/g which is the highest TPC in this study. Flavonoids were not detected in *P*. *Ostreatus*in the findings of Matilla et al [90] and Chowdhury et al [91]. It is reported by USDA that mushrooms are considered as non-sources of flavonoids and our finding agrees with the abovementioned reports as the highest TFC observed is 0.744 mg QAE/g.

The DPPH and ABTS assays are known for their reproducibility; ease of application and low cost [92] The ABTS radical scavenging activity of is a more sensitive radical for assessment of antioxidant activity. The discoloration of ABTS radical cation shows the ability of the antioxidants to deactivate these radical species by donating either electrons or hydrogen atoms [93]. As shown in Table 5, the average scavenging activity of ABTS radical is 6.805 mg/ml which is slightly higher than *S*. *commune* BPSM01 with an IC_50_ of 3.25 mg/ml which is the most significant antioxidant activity in the methanolic extract of these mushrooms.

## Conclusions

The macrofungal diversity from two protected forest areas in India was undertaken and their antimicrobial potential was investigated. For the first time 231 wild macrofungi were collected and documented from the selected locations. The significant antifungal and antibacterial activity was found in *Microporus* sp. and *Schizophyllum commune* respectively. Further testing of these strains for the discovery of novel and efficient antimicrobials should be considered. *Microporus* sp. strain BPSM 33 showed the highest antagonistic potential against the phytopathogen *F*. *oxysporum* with 85.71% of inhibition and could be used for the development of biocontrol agent against *F*. *oxysporum*. Furthermore, the genetic variation among the potential strains showing antimicrobial potential was considered by using RAPD and ISSR molecular markers. We reported that the selected markers can be used for assessing genetic diversity of wild mushrooms, considering both marker systems gave similar results in the present study. We also conclude that the wild edible mushroom strains does not have only nutritional values but also can be used as an accessible source of natural antioxidants.
